# (±)-1,2-Bis(*N*′-benzoyl­thio­ureido)cyclo­hexa­ne

**DOI:** 10.1107/S1600536811014991

**Published:** 2011-04-29

**Authors:** Juliana Jumal, Abdul Razak Ibrahim, Bohari M. Yamin

**Affiliations:** a Faculty of Science and Technology, Universiti Sains Islam Malaysia, 71800 Nilai N. Sembilan, Malaysia; b X-ray Crystallography Unit, School of Physics, Universiti Sains Malaysia, USM 11800, Penang, Malaysia; c School of Chemical Sciences and Food Technology, Universiti Kebangsaan Malaysia, UKM 43500, Bangi Selangor, Malaysia

## Abstract

In the title compound, C_22_H_24_N_4_O_2_S_2_, the two thio­urea segments of the side-arm groups are inclined at a dihedral angle of 73.09 (9)°. The central cyclo­hexane bridge adopts a chair conformation. The mol­ecule is stabilized by N—H⋯O intra­molecular hydrogen bonds forming *S*(6) rings, and N—H⋯O and N—H⋯S inter­molecular hydrogen bonds forming infinite chains developing parallel to the *b* axis.

## Related literature

For related structures, see: Yusof *et al.* (2008 ▶); Thiam *et al.* (2008 ▶). For bond-length data, see Allen *et al.* (1987 ▶). For a description of hydrogen-bonding patterns, see: Etter *et al.* (1990 ▶); Bernstein *et al.* (1995 ▶).
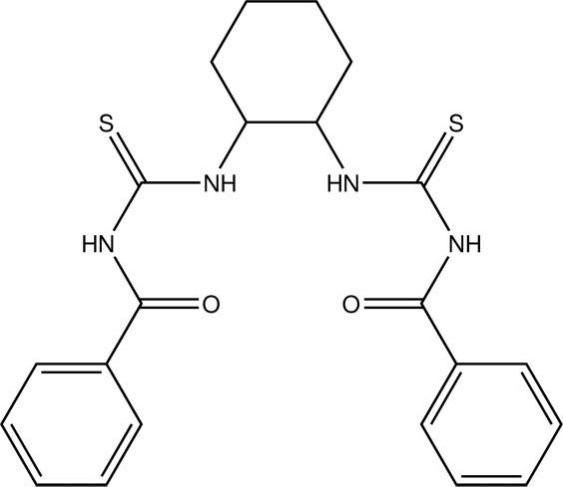


## Experimental

### 

#### Crystal data

C_22_H_24_N_4_O_2_S_2_
*M* *_r_* = 440.57
Monoclinic, 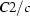

*a* = 19.725 (6) Å
*b* = 11.054 (3) Å
*c* = 20.700 (5) Å
β = 91.252 (9)°
*V* = 4512 (2) Å^3^
*Z* = 8
Mo *K*α radiation
μ = 0.26 mm^−1^
*T* = 298 K
0.45 × 0.39 × 0.20 mm

#### Data collection

Bruker SMART APEX CCD area-detector diffractometer
Absorption correction: multi-scan (*SADABS*; Bruker, 2000 ▶) *T*_min_ = 0.891, *T*_max_ = 0.950
16892 measured reflections
4212 independent reflections
3334 reflections with *I* > 2σ(*I*)
*R*_int_ = 0.043

#### Refinement

*R*[*F* ^2^ > 2σ(*F* ^2^)] = 0.062
*wR*(*F* ^2^) = 0.145
*S* = 1.11
4212 reflections
272 parameters
H-atom parameters constrained
Δρ_max_ = 0.37 e Å^−3^
Δρ_min_ = −0.21 e Å^−3^

### 

Data collection: *SMART* (Bruker, 2000 ▶); cell refinement: *SAINT* (Bruker, 2000 ▶); data reduction: *SAINT*; program(s) used to solve structure: *SHELXTL* (Sheldrick, 2008 ▶); program(s) used to refine structure: *SHELXTL*; molecular graphics: *ORTEPIII* (Burnett & Johnson, 1996 ▶), *ORTEP-3 for Windows* (Farrugia, 1997 ▶) and *PLATON* (Spek, 2009 ▶); software used to prepare material for publication: *SHELXTL*, *PARST* (Nardelli, 1995 ▶) and *PLATON*.

## Supplementary Material

Crystal structure: contains datablocks global, I. DOI: 10.1107/S1600536811014991/dn2675sup1.cif

Structure factors: contains datablocks I. DOI: 10.1107/S1600536811014991/dn2675Isup2.hkl

Supplementary material file. DOI: 10.1107/S1600536811014991/dn2675Isup3.cml

Additional supplementary materials:  crystallographic information; 3D view; checkCIF report

## Figures and Tables

**Table 1:** Hydrogen-bond geometry (Å, °)

*D*—H⋯*A*	*D*—H	H⋯*A*	*D*⋯*A*	*D*—H⋯*A*
N2—H2*A*⋯O1	0.86	2.01	2.677 (3)	134
N3—H3*A*⋯O2	0.86	1.96	2.645 (3)	136
N1—H1*A*⋯O2^i^	0.86	2.26	3.077 (3)	159
N4—H4*A*⋯S2^ii^	0.86	2.56	3.405 (3)	166

Symmetry codes: (i) 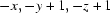; (ii) 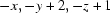.

**Fig. 1.**
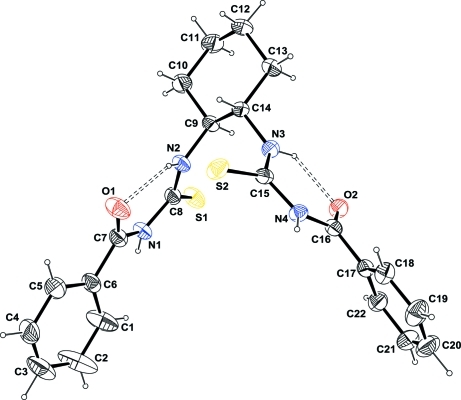
The asymmetric unit of (I) with the atom-labelling scheme. Displacement ellipsoids are drawn at the 30% probability level. H atoms are represented as small spheres of arbitrary radii. Hydrogen bonds are shown as dashed lines.

**Fig. 2.**
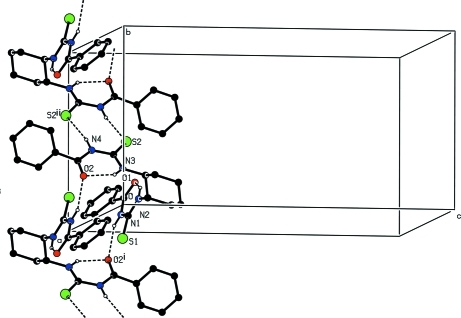
Partial packing view of compound ( I ), showing the formation of chains along the b axis built from hydrogen bonds, and the formation of R 42(18) and R22(8) rings. For the sake of clarity, H atoms not involved in hydrogen bonding have been omitted.[Symmetry codes: (i) -x, -y+1, -z+1; (ii) -x, -y+2, -z+1]

## supplementary crystallographic information

### Comment

The title compound, (I) is similar to 1,2-bis[*N*'-(2,2-dimethylpropionyl) thioureido]cyclohexane (Yusof *et al.*, 2008) except the two side arms are benzoylthioureido (Fig. 1) groups instead of 2,2-dimethylpropionylthioureido.The bond lengths and angles are in normal ranges (Allen  *et al.*, 1987) and comparable to those in 1,2-bis[*N*'-(2,2-dimethylpropionyl)thioureido]cycylohexane and 1,2-bis (*N*'-benzoylthioureido)benzene (Thiam *et al.*, 2008). However, the dihedral angle between the thiourea groups of 73.09 (9)° is slightly smaller compare to 78.55 (7)° in the propionylthioureido analog.

Both thiourea moieties, S1/N2/C7/C8/C9 and S2/N3/N4/C14/C15 are planar with maximum deviation of 0.017 (3)Å for C9 atom from the least square plane. There are two intramolecular hydrogen bonds N2—H2A..O1 and N3—H3A···O2 forming two pseudo-six membered rings S(6) (Etter *et al.*, 1990; Bernstein *et al.*, 1995) O1···H2A—N2—C8—N1—C7 and O2..H3A—N3—C15—N4—C16 respectively (Table 1). In the crystal structure, the molecules are linked by intermolecular hydrogen bonds N1—H1A···O2 forming a R^4^_2_(18) graph set motif and N4—H4A···S2 forming a R^2^_2_(8) motif (Etter *et al.*, 1990; Bernstein *et al.*, 1995). These intermolecular interactions result in the formation of chains extending along the *b* axis (Fig.2; Table 1).

### Experimental

A solution of benzoylisothiocyanate (3.26 g, 0.02 mol) in 30 ml acetone was added into a flask containing 30 ml acetone solution of 1,2-diamino cyclohexane (1.14 g, 0.01 mol).The mixture was refluxed for 1 h. Then, the solution was filtered-off and left to evaporate at room temperature. The colourless solid was obtained after one day of evaporation(yield 81%, m.p 495.3–497.3 K)

### Refinement

H atoms on the parent carbon atoms were positioned geometrically with C—H= 0.96–0.98 Å and constrained to ride on their parent atoms with *U*_iso_(H)= *xU*_eq_(parent atom) where *x*=1.5 for CH_3_ group and 1.2 for CH_2_ and CH groups.

### Crystal data

**Table:**

C_22_H_24_N_4_O_2_S_2_	*F*(000) = 1856
*M**_r_* = 440.57	*D*_x_ = 1.297 Mg m^−^^3^
Monoclinic, *C*2/*c*	Melting point = 495.3–497.3 K
Hall symbol: -C 2yc	Mo *K*α radiation, λ = 0.71073 Å
*a* = 19.725 (6) Å	Cell parameters from 4375 reflections
*b* = 11.054 (3) Å	θ = 2.0–25.2°
*c* = 20.700 (5) Å	µ = 0.26 mm^−^^1^
β = 91.252 (9)°	*T* = 298 K
*V* = 4512 (2) Å^3^	Block, colourless
*Z* = 8	0.45 × 0.39 × 0.20 mm

### Data collection

**Table:**

Bruker SMART APEX CCD area-detector diffractometer	4212 independent reflections
Radiation source: fine-focus sealed tube	3334 reflections with *I* > 2σ(*I*)
graphite	*R*_int_ = 0.043
Detector resolution: 83.66 pixels mm^-1^	θ_max_ = 25.5°, θ_min_ = 2.0°
ω scans	*h* = −23→23
Absorption correction: multi-scan (*SADABS*; Bruker, 2000)	*k* = −13→13
*T*_min_ = 0.891, *T*_max_ = 0.950	*l* = −24→25
16892 measured reflections	

### Refinement

**Table:**

Refinement on *F*^2^	Primary atom site location: structure-invariant direct methods
Least-squares matrix: full	Secondary atom site location: difference Fourier map
*R*[*F*^2^ > 2σ(*F*^2^)] = 0.062	Hydrogen site location: inferred from neighbouring sites
*wR*(*F*^2^) = 0.145	H-atom parameters constrained
*S* = 1.11	*w* = 1/[σ^2^(*F*_o_^2^) + (0.0596*P*)^2^ + 4.3773*P*] where *P* = (*F*_o_^2^ + 2*F*_c_^2^)/3
4212 reflections	(Δ/σ)_max_ = 0.001
272 parameters	Δρ_max_ = 0.37 e Å^−^^3^
0 restraints	Δρ_min_ = −0.21 e Å^−^^3^

### Special details

**Table:**

Geometry. All e.s.d.'s (except the e.s.d. in the dihedral angle between two l.s. planes) are estimated using the full covariance matrix. The cell e.s.d.'s are taken into account individually in the estimation of e.s.d.'s in distances, angles and torsion angles; correlations between e.s.d.'s in cell parameters are only used when they are defined by crystal symmetry. An approximate (isotropic) treatment of cell e.s.d.'s is used for estimating e.s.d.'s involving l.s. planes.
Refinement. Refinement of *F*^2^ against ALL reflections. The weighted *R*-factor *wR* and goodness of fit *S* are based on *F*^2^, conventional *R*-factors *R* are based on *F*, with *F* set to zero for negative *F*^2^. The threshold expression of *F*^2^ > σ(*F*^2^) is used only for calculating *R*-factors(gt) *etc*. and is not relevant to the choice of reflections for refinement. *R*-factors based on *F*^2^ are statistically about twice as large as those based on *F*, and *R*- factors based on ALL data will be even larger.

### Fractional atomic coordinates and isotropic or equivalent isotropic displacement parameters (Å^2^)

**Table:**

	*x*	*y*	*z*	*U*_iso_*/*U*_eq_	
S1	0.02397 (4)	0.38387 (8)	0.42308 (5)	0.0633 (3)	
S2	0.02746 (4)	0.92533 (8)	0.41333 (4)	0.0586 (3)	
O1	−0.09838 (10)	0.7174 (2)	0.36607 (12)	0.0702 (7)	
O2	0.14844 (9)	0.71111 (18)	0.56241 (9)	0.0492 (5)	
N1	−0.07970 (10)	0.5330 (2)	0.41058 (11)	0.0468 (6)	
H1A	−0.1004	0.4730	0.4279	0.056*	
N2	0.02222 (10)	0.6069 (2)	0.37479 (11)	0.0454 (6)	
H2A	0.0001	0.6723	0.3664	0.054*	
N3	0.12087 (11)	0.7590 (2)	0.43944 (10)	0.0450 (6)	
H3A	0.1456	0.7217	0.4678	0.054*	
N4	0.08148 (11)	0.86601 (19)	0.52667 (10)	0.0410 (5)	
H4A	0.0591	0.9281	0.5394	0.049*	
C1	−0.21028 (19)	0.5515 (3)	0.4688 (2)	0.0898 (13)	
H1B	−0.1791	0.4990	0.4881	0.108*	
C2	−0.2756 (2)	0.5544 (4)	0.4898 (3)	0.124 (2)	
H2B	−0.2884	0.5035	0.5231	0.149*	
C3	−0.32249 (19)	0.6311 (5)	0.4623 (3)	0.1007 (17)	
H3B	−0.3668	0.6324	0.4767	0.121*	
C4	−0.3036 (2)	0.7045 (5)	0.4142 (2)	0.1030 (17)	
H4B	−0.3352	0.7564	0.3951	0.124*	
C5	−0.23709 (17)	0.7038 (4)	0.39271 (18)	0.0871 (13)	
H5A	−0.2245	0.7565	0.3601	0.104*	
C6	−0.19050 (14)	0.6265 (3)	0.41907 (15)	0.0523 (8)	
C7	−0.11937 (14)	0.6315 (3)	0.39623 (14)	0.0477 (7)	
C8	−0.01047 (13)	0.5160 (3)	0.40108 (13)	0.0440 (6)	
C9	0.09410 (13)	0.6023 (3)	0.35922 (13)	0.0446 (6)	
H9A	0.1160	0.5420	0.3874	0.054*	
C10	0.10317 (15)	0.5628 (3)	0.28926 (15)	0.0606 (8)	
H10A	0.0819	0.4846	0.2825	0.073*	
H10B	0.0810	0.6205	0.2605	0.073*	
C11	0.17797 (17)	0.5547 (4)	0.27354 (17)	0.0723 (10)	
H11A	0.1993	0.4919	0.2997	0.087*	
H11B	0.1827	0.5330	0.2285	0.087*	
C12	0.21279 (16)	0.6735 (4)	0.28648 (16)	0.0701 (10)	
H12A	0.1947	0.7342	0.2570	0.084*	
H12B	0.2609	0.6652	0.2786	0.084*	
C13	0.20305 (14)	0.7155 (4)	0.35590 (15)	0.0631 (9)	
H13A	0.2238	0.7944	0.3619	0.076*	
H13B	0.2255	0.6594	0.3853	0.076*	
C14	0.12781 (12)	0.7232 (3)	0.37194 (12)	0.0434 (6)	
H14A	0.1061	0.7845	0.3442	0.052*	
C15	0.07964 (13)	0.8440 (2)	0.46047 (12)	0.0419 (6)	
C16	0.11438 (12)	0.8012 (2)	0.57403 (13)	0.0398 (6)	
C17	0.10748 (12)	0.8470 (3)	0.64079 (13)	0.0419 (6)	
C18	0.10957 (15)	0.9688 (3)	0.65517 (14)	0.0526 (7)	
H18A	0.1143	1.0253	0.6223	0.063*	
C19	0.10464 (18)	1.0065 (3)	0.71809 (17)	0.0713 (10)	
H19A	0.1066	1.0886	0.7278	0.086*	
C20	0.09689 (18)	0.9240 (4)	0.76659 (17)	0.0742 (10)	
H20A	0.0933	0.9504	0.8090	0.089*	
C21	0.09432 (17)	0.8037 (4)	0.75319 (15)	0.0673 (9)	
H21A	0.0884	0.7482	0.7863	0.081*	
C22	0.10055 (15)	0.7639 (3)	0.69031 (14)	0.0546 (8)	
H22A	0.1001	0.6815	0.6813	0.065*	

### Atomic displacement parameters (Å^2^)

**Table:**

	*U*^11^	*U*^22^	*U*^33^	*U*^12^	*U*^13^	*U*^23^
S1	0.0446 (4)	0.0520 (5)	0.0936 (7)	0.0080 (3)	0.0057 (4)	0.0092 (4)
S2	0.0634 (5)	0.0719 (6)	0.0406 (4)	0.0246 (4)	0.0012 (3)	0.0038 (4)
O1	0.0475 (12)	0.0653 (15)	0.0982 (18)	0.0125 (11)	0.0131 (12)	0.0236 (13)
O2	0.0491 (11)	0.0522 (12)	0.0463 (11)	0.0115 (9)	−0.0001 (9)	−0.0018 (9)
N1	0.0325 (11)	0.0479 (13)	0.0602 (15)	0.0014 (10)	0.0076 (10)	0.0023 (11)
N2	0.0320 (11)	0.0500 (13)	0.0544 (14)	0.0050 (10)	0.0054 (10)	0.0016 (11)
N3	0.0399 (12)	0.0545 (14)	0.0405 (13)	0.0060 (11)	0.0009 (10)	−0.0002 (11)
N4	0.0438 (12)	0.0408 (12)	0.0387 (12)	0.0064 (10)	0.0041 (9)	0.0007 (10)
C1	0.067 (2)	0.060 (2)	0.144 (4)	0.0126 (18)	0.050 (2)	0.012 (2)
C2	0.086 (3)	0.073 (3)	0.217 (6)	0.002 (2)	0.090 (4)	0.002 (3)
C3	0.045 (2)	0.112 (4)	0.147 (5)	−0.008 (2)	0.030 (2)	−0.065 (3)
C4	0.051 (2)	0.175 (5)	0.083 (3)	0.042 (3)	−0.009 (2)	−0.032 (3)
C5	0.053 (2)	0.141 (4)	0.067 (2)	0.036 (2)	0.0017 (17)	0.002 (2)
C6	0.0376 (15)	0.0555 (18)	0.064 (2)	0.0017 (13)	0.0041 (13)	−0.0204 (15)
C7	0.0389 (14)	0.0502 (18)	0.0541 (18)	0.0014 (13)	0.0022 (13)	−0.0044 (14)
C8	0.0365 (13)	0.0499 (16)	0.0456 (16)	0.0021 (12)	0.0004 (11)	−0.0064 (13)
C9	0.0335 (13)	0.0547 (17)	0.0459 (16)	0.0058 (12)	0.0061 (11)	−0.0004 (13)
C10	0.0539 (18)	0.072 (2)	0.0557 (19)	0.0005 (16)	0.0075 (14)	−0.0163 (16)
C11	0.060 (2)	0.100 (3)	0.057 (2)	0.018 (2)	0.0176 (16)	−0.0145 (19)
C12	0.0414 (16)	0.111 (3)	0.059 (2)	0.0034 (18)	0.0204 (14)	−0.005 (2)
C13	0.0391 (16)	0.093 (3)	0.0574 (19)	−0.0060 (16)	0.0096 (13)	−0.0071 (18)
C14	0.0352 (13)	0.0594 (18)	0.0358 (14)	0.0029 (12)	0.0063 (11)	−0.0006 (13)
C15	0.0370 (13)	0.0480 (16)	0.0411 (15)	−0.0013 (12)	0.0077 (11)	0.0026 (13)
C16	0.0327 (13)	0.0414 (15)	0.0453 (15)	−0.0023 (11)	0.0028 (11)	0.0035 (12)
C17	0.0343 (13)	0.0500 (16)	0.0413 (15)	0.0019 (12)	−0.0009 (11)	0.0017 (13)
C18	0.0573 (17)	0.0518 (18)	0.0485 (17)	0.0038 (14)	−0.0049 (13)	−0.0018 (14)
C19	0.089 (3)	0.064 (2)	0.061 (2)	0.0148 (19)	−0.0099 (18)	−0.0170 (18)
C20	0.082 (2)	0.096 (3)	0.0451 (19)	0.016 (2)	0.0042 (17)	−0.018 (2)
C21	0.073 (2)	0.088 (3)	0.0418 (18)	−0.0025 (19)	0.0034 (15)	0.0087 (17)
C22	0.0590 (18)	0.0589 (19)	0.0457 (17)	−0.0045 (15)	−0.0003 (14)	0.0005 (15)

### Geometric parameters (Å, °)

**Table:**

S1—C8	1.670 (3)	C9—C14	1.513 (4)
S2—C15	1.666 (3)	C9—C10	1.527 (4)
O1—C7	1.215 (3)	C9—H9A	0.9800
O2—C16	1.228 (3)	C10—C11	1.521 (4)
N1—C7	1.370 (4)	C10—H10A	0.9700
N1—C8	1.397 (3)	C10—H10B	0.9700
N1—H1A	0.8600	C11—C12	1.504 (5)
N2—C8	1.318 (3)	C11—H11A	0.9700
N2—C9	1.462 (3)	C11—H11B	0.9700
N2—H2A	0.8600	C12—C13	1.526 (4)
N3—C15	1.323 (3)	C12—H12A	0.9700
N3—C14	1.462 (3)	C12—H12B	0.9700
N3—H3A	0.8600	C13—C14	1.530 (4)
N4—C16	1.366 (3)	C13—H13A	0.9700
N4—C15	1.392 (3)	C13—H13B	0.9700
N4—H4A	0.8600	C14—H14A	0.9800
C1—C2	1.369 (5)	C16—C17	1.481 (4)
C1—C6	1.385 (5)	C17—C18	1.379 (4)
C1—H1B	0.9300	C17—C22	1.386 (4)
C2—C3	1.369 (7)	C18—C19	1.373 (4)
C2—H2B	0.9300	C18—H18A	0.9300
C3—C4	1.342 (7)	C19—C20	1.367 (5)
C3—H3B	0.9300	C19—H19A	0.9300
C4—C5	1.394 (5)	C20—C21	1.359 (5)
C4—H4B	0.9300	C20—H20A	0.9300
C5—C6	1.360 (5)	C21—C22	1.382 (4)
C5—H5A	0.9300	C21—H21A	0.9300
C6—C7	1.491 (4)	C22—H22A	0.9300
			
C7—N1—C8	129.2 (2)	C12—C11—C10	110.6 (3)
C7—N1—H1A	115.4	C12—C11—H11A	109.5
C8—N1—H1A	115.4	C10—C11—H11A	109.5
C8—N2—C9	123.4 (2)	C12—C11—H11B	109.5
C8—N2—H2A	118.3	C10—C11—H11B	109.5
C9—N2—H2A	118.3	H11A—C11—H11B	108.1
C15—N3—C14	125.3 (2)	C11—C12—C13	111.5 (3)
C15—N3—H3A	117.4	C11—C12—H12A	109.3
C14—N3—H3A	117.4	C13—C12—H12A	109.3
C16—N4—C15	128.1 (2)	C11—C12—H12B	109.3
C16—N4—H4A	116.0	C13—C12—H12B	109.3
C15—N4—H4A	116.0	H12A—C12—H12B	108.0
C2—C1—C6	120.2 (4)	C12—C13—C14	111.3 (2)
C2—C1—H1B	119.9	C12—C13—H13A	109.4
C6—C1—H1B	119.9	C14—C13—H13A	109.4
C1—C2—C3	121.0 (5)	C12—C13—H13B	109.4
C1—C2—H2B	119.5	C14—C13—H13B	109.4
C3—C2—H2B	119.5	H13A—C13—H13B	108.0
C4—C3—C2	119.1 (4)	N3—C14—C9	110.8 (2)
C4—C3—H3B	120.5	N3—C14—C13	109.5 (2)
C2—C3—H3B	120.5	C9—C14—C13	109.8 (2)
C3—C4—C5	120.8 (4)	N3—C14—H14A	108.9
C3—C4—H4B	119.6	C9—C14—H14A	108.9
C5—C4—H4B	119.6	C13—C14—H14A	108.9
C6—C5—C4	120.5 (4)	N3—C15—N4	116.4 (2)
C6—C5—H5A	119.8	N3—C15—S2	124.6 (2)
C4—C5—H5A	119.8	N4—C15—S2	119.00 (19)
C5—C6—C1	118.4 (3)	O2—C16—N4	122.5 (2)
C5—C6—C7	118.8 (3)	O2—C16—C17	121.5 (2)
C1—C6—C7	122.7 (3)	N4—C16—C17	116.0 (2)
O1—C7—N1	122.3 (3)	C18—C17—C22	119.4 (3)
O1—C7—C6	121.7 (3)	C18—C17—C16	122.2 (3)
N1—C7—C6	116.0 (3)	C22—C17—C16	118.4 (3)
N2—C8—N1	116.4 (2)	C19—C18—C17	119.9 (3)
N2—C8—S1	125.4 (2)	C19—C18—H18A	120.0
N1—C8—S1	118.2 (2)	C17—C18—H18A	120.0
N2—C9—C14	110.8 (2)	C20—C19—C18	120.4 (3)
N2—C9—C10	110.7 (2)	C20—C19—H19A	119.8
C14—C9—C10	110.9 (2)	C18—C19—H19A	119.8
N2—C9—H9A	108.1	C21—C20—C19	120.4 (3)
C14—C9—H9A	108.1	C21—C20—H20A	119.8
C10—C9—H9A	108.1	C19—C20—H20A	119.8
C11—C10—C9	110.8 (2)	C20—C21—C22	120.0 (3)
C11—C10—H10A	109.5	C20—C21—H21A	120.0
C9—C10—H10A	109.5	C22—C21—H21A	120.0
C11—C10—H10B	109.5	C21—C22—C17	119.8 (3)
C9—C10—H10B	109.5	C21—C22—H22A	120.1
H10A—C10—H10B	108.1	C17—C22—H22A	120.1
			
C6—C1—C2—C3	−0.2 (7)	C15—N3—C14—C13	132.9 (3)
C1—C2—C3—C4	−0.2 (8)	N2—C9—C14—N3	58.4 (3)
C2—C3—C4—C5	−0.5 (7)	C10—C9—C14—N3	−178.2 (2)
C3—C4—C5—C6	1.5 (7)	N2—C9—C14—C13	179.4 (2)
C4—C5—C6—C1	−1.8 (6)	C10—C9—C14—C13	−57.2 (3)
C4—C5—C6—C7	−178.0 (4)	C12—C13—C14—N3	177.7 (3)
C2—C1—C6—C5	1.2 (6)	C12—C13—C14—C9	55.9 (4)
C2—C1—C6—C7	177.2 (4)	C14—N3—C15—N4	−178.8 (2)
C8—N1—C7—O1	7.3 (5)	C14—N3—C15—S2	0.5 (4)
C8—N1—C7—C6	−173.5 (3)	C16—N4—C15—N3	−8.8 (4)
C5—C6—C7—O1	16.3 (5)	C16—N4—C15—S2	171.9 (2)
C1—C6—C7—O1	−159.7 (3)	C15—N4—C16—O2	0.5 (4)
C5—C6—C7—N1	−162.9 (3)	C15—N4—C16—C17	179.7 (2)
C1—C6—C7—N1	21.1 (4)	O2—C16—C17—C18	138.7 (3)
C9—N2—C8—N1	−177.5 (2)	N4—C16—C17—C18	−40.5 (3)
C9—N2—C8—S1	2.1 (4)	O2—C16—C17—C22	−39.6 (4)
C7—N1—C8—N2	0.2 (4)	N4—C16—C17—C22	141.3 (3)
C7—N1—C8—S1	−179.4 (2)	C22—C17—C18—C19	−0.1 (4)
C8—N2—C9—C14	−143.0 (3)	C16—C17—C18—C19	−178.4 (3)
C8—N2—C9—C10	93.5 (3)	C17—C18—C19—C20	−0.7 (5)
N2—C9—C10—C11	−178.4 (3)	C18—C19—C20—C21	0.3 (5)
C14—C9—C10—C11	58.1 (4)	C19—C20—C21—C22	0.9 (5)
C9—C10—C11—C12	−56.7 (4)	C20—C21—C22—C17	−1.8 (5)
C10—C11—C12—C13	55.7 (4)	C18—C17—C22—C21	1.4 (4)
C11—C12—C13—C14	−55.7 (4)	C16—C17—C22—C21	179.7 (3)
C15—N3—C14—C9	−105.9 (3)		

### Hydrogen-bond geometry (Å, °)

**Table:**

*D*—H···*A*	*D*—H	H···*A*	*D*···*A*	*D*—H···*A*
N2—H2A···O1	0.86	2.01	2.677 (3)	134
N3—H3A···O2	0.86	1.96	2.645 (3)	136
N1—H1A···O2^i^	0.86	2.26	3.077 (3)	159
N4—H4A···S2^ii^	0.86	2.56	3.405 (3)	166

Symmetry codes:  (i) −*x*, −*y*+1, −*z*+1; (ii) −*x*, −*y*+2, −*z*+1.
